# Green Synthesis of Silver Nanoparticles (AgNPs) of *Angelica Gigas* Fabricated by Hot-Melt Extrusion Technology for Enhanced Antifungal Effects

**DOI:** 10.3390/ma15207231

**Published:** 2022-10-17

**Authors:** Suji Ryu, Seoul-Hee Nam, Jong-Suep Baek

**Affiliations:** 1 Department of Bio-Health Convergence, Kangwon National University, Chuncheon 24341, Korea; 2 Department of Dental Hygiene, Kangwon National University, Samcheok 25949, Korea; 3 Department of Herbal Medicine Resource, Kangwon National University, Samcheok 25949, Korea

**Keywords:** *Angelica gigas* Nakai, silver nanoparticle, green synthesis, antifungal, *Candida albicans*

## Abstract

Green synthesis for synthesizing silver nanoparticles (AgNPs) has been suggested as an environmentally friendly alternative to conventional physical/chemical methods. In this study, we report the green synthesis of AgNPs using a hot-melt extrusion-processed *Angelica gigas* Nakai (AGN) (HME-AGN) extract as a reducing agent to increase the water solubility of the active ingredient compared to the existing AGN. The mixture of the AGN extract and AgNO_3_ at about 420 nm could not confirm the formation of AgNPs. The synthesis of AgNPs was found to be most advantageous at 60 °C when the mixing ratio of the HME-AGN extract was 9:1 (AgNO_3_–extract, *v*/*v*) using 3 mM AgNO_3_. The physicochemical properties of the optimized AgNPs were characterized by UV–Vis spectrophotometer, dynamic light scattering (DLS), zeta potential, transmission electron microscopy (TEM), energy dispersive spectroscopy (EDS), Fourier-transform infrared spectroscopy (FT-IR), and X-ray diffractometer (XRD). DLS showed the particle size average of 102.3 ± 1.35 nm and polydispersity index (PDI) value of 0.314 ± 0.01. The particle surface charge was −35 ± 0.79 mV, confirming the stability of the particles. The particle shape was spherical, as shown through TEM analysis, and the presence of silver ions was confirmed through the EDS results. FT-IR data showed functional groups of biomolecules of the extract involved in the synthesis of AgNPs. The face-centered cubic (FCC) lattice of AgNPs was confirmed in the XRD pattern. The AgNPs had an effective antifungal activity against *Candida albicans* (*C. albicans*) that was better than that of the HME-AGN extract. In conclusion, this study suggests that the synthesis of AgNPs was improved by using the HME-AGN extract with increased water solubility through HME. In addition, it was suggested that the synthesized AgNPs can be used as an improved antifungal agent compared with the HME-AGN extract with antifungal activity.

## 1. Introduction

In the last few years, nanoparticles have been heavily used in cosmetics, medicines, and food preservatives that come into direct contact with humans [1,2,3]. Silver nanoparticles (AgNPs) were found to be very effective in terms of their antibacterial [4] and antifungal activity [5]. *Candida albicans* (*C. albicans*) is the yeast most involved in human infection and is still considered an important human fungal pathogen [6,7]. A large number of studies have demonstrated the anti-*C. albicans* activity using AgNPs [8,9]. Generally, AgNPs are synthesized via several chemical and physical approaches. Physical methods such as ball milling, flame sonication, radiation, electric arc discharge, and pyrolysis require high energy consumption and a high cost [10]. In addition, the chemical method needs solvents that are harmful for the environment [11]. The green synthesis method is an environmentally friendly process that does not require high energy and toxic chemicals and has been proposed as an alternative to the aforementioned physical and chemical manufacturing methods [12,13]. In recent years, synthetic methods to obtain nanomaterials, employing biological materials such as algae [14], plant extracts [15], fungi [16], and bacteria [17], have been introduced as facile and feasible alternatives to the more complex chemical synthetic procedures [18]. The method of synthesizing AgNPs using plant extracts has advantages in that the material is more widely distributed, readily scaled-up, easily available, safe to handle, and less expensive than other green synthesis methods [19]. The extracts used for AgNPs’ synthesis perform as reducing agents and stabilizing agents in the synthesis process [20].

*Angelica gigas* Nakai (AGN) is a biennial or short-lived perennial plant [21]. In Asian countries, the root of AGN is one of the most used herbal medicines [22]. The active ingredients of Angelica are different according to its geographical origins, such as Korea, Japan, and China, so the biological efficacy varies accordingly. AGN is a Korean Angelica, unlike Chinese and Japanese Angelica, and contains a large amount of furanocoumarin-based decursin and decursinol angelate [23]. In addition, AGN is known to have various pharmacologically active properties such as antioxidant [24], antibacterial, anticancer [25], and antiinflammatory [26].

Hot-melt extrusion (HME) is one of the technologies for processing food and drugs through shear and friction techniques using screws under high-temperature and high-pressure conditions [27]. HME is being investigated for its ability to produce solid dispersions to improve the oral bioavailability of poorly soluble drugs [28,29]. HME technology has recently been introduced to natural product processing and has been used to improve water solubility and the bioavailability of hydrophobic compounds using appropriate excipients [30]. As reported in previous studies, the improved water solubility of the active ingredient of AGN through HME and enhanced antifungal activity against *C. albicans* have been demonstrated [31].

This study aimed to synthesize AgNPs using an HME-AGN extract in order to propose a new route to synthesize AgNPs that can be used as an antifungal agent. It was investigated that an HME-AGN extract with increased water solubility through HME can be used as an improved reducing agent compared with an AGN extract. Furthermore, the antifungal activity of AgNPs synthesized using extracts of natural products processed with HME was investigated for the first time.

## 2. Materials and Methods

### 2.1. Materials

Silver nitrate (AgNO_3_) was purchased from Daejung chemical (Siheung, Korea). Phosphate-buffered saline (PBS, Wegene) was used without further purification system. All other ingredients, solvents, and reagents were of analytical grade.

### 2.2. Preparation of the Plant Extract

HME-AGN was prepared through HME (STS-25HS tween screw extruder, Hankook E.M. Ltd., Pyoung-Taek, Korea) by adding AGN with screw conditions of 15 bar and 150 rpm at 100 °C. HME-AGN extract was prepared by weighing 1 g of dried HME-AGN powder in 50 mL of distilled water and sonicated in 40 °C water bath for 1 h. Then, the extract was centrifuged at 3000 rpm and the supernatant was filtered by Whatman filter paper (No. 6) and stored at 4 °C for further studies.

### 2.3. Synthesis of AgNPs

The optimization of the AgNPs was performed by modifying various parameters. Different concentrations of AgNO_3_ i.e., 1, 2, and 3 mM, and leaf extract, i.e., 0.2, 0.4, 0.6, 0.8, and 1.0 mL, different temperatures, i.e., 20, 40, 60, and 80 °C, and different reaction times, i.e., 1, 2, 4, 18, and 24 h were employed to standardize the optimum conditions. The sample was analyzed with a UV–visible spectrophotometer (Thermo Fisher Scientific, Waltham, MA, USA) to confirm the synthesis of AgNPs. Synthesized AgNPs in optimized conditions were centrifuged at 13,000× *g* rpm for 10 min. The centrifugation process was repeated 3 times by dispersing the pellet in distilled water for removing the organic matter of HME-AGN extract. Collected AgNPs were dried in a freeze dryer and stored in dark conditions at room temperature.

### 2.4. Visual Inspection

Through the color change after mixing plant extract with AgNO_3_, the reduction of Ag^+^ to Ag^0^ was confirmed [32]. To confirm the color change through the synthesis of AgNPs using HME-AGN extract, HME-AGN extract and AgNO_3_ solution were mixed in a ratio of 1:9, and the color change was compared according to 5 min, 1 h, and 6 h.

### 2.5. Characterization of AgNPs

The synthesis of AgNPs was investigated by UV–Vis spectrophotometer (BioMate 160, Thermo Fisher Scientific, Waltham, MA, USA). Absorbance was confirmed at a wavelength of 300–700 nm using a cuvette cell containing 1 mL of sample. AgNPs exhibit strong electromagnetic wave absorption in the visible range on account of the surface plasmon resonance (SPR) [33,34]. The particle size, polydispersity index (PDI), and zeta potential were determined by using a Mastersizer 2000 (Malvern Instruments, Malvern, UK). AgNP sample was loaded on the copper grid to observe the size and morphology using the TEM equipped with energy dispersive spectroscopy (EDS) capability (JEM-2100F, JEOL, Tokyo, Japan). XRD pattern of AgNPs was investigated using an X-ray diffractometer (X’pert-pro MPD, PAN analytical, Netherland) at a 2θ degree angle ranging from 5 to 80°. The chemical compositions of plant extract and the synthesized AgNPs were studied using an FT-IR spectrometer (Nicolet iN10, Thermo Fisher Scientific, Waltham, MA, USA) in the range of 400–4000 cm^−1^.

### 2.6. Antifungal Effect

The antifungal activity was investigated based on previous studies [31]. The antifungal activity of AgNPs was tested using the colony-forming unit (CFU) method. The strain of *C. albicans* (KCTC 7965/ATCC 10231) was used as the test organism. The strain of *C. albicans* was grown in yeast mold (YM) broth (Difco, Detroit, MI, USA) and cultivated overnight in liquid media incubated at 37 °C. The optical density (OD) measurements of cell growth were tested by measuring the absorbance at 660 nm. The cell was diluted in phosphate-buffered saline (PBS) to a final concentration of 5 × 10^5^ CFU/mL. Next, 100 μL of cultured *C. albicans* (5 × 10^5^ CFUs/mL) was inoculated into a 24-microwell plate containing YM broth in which HME-AGN extract and AgNPs were added at each concentration (0.01, 0.1, 1, 5, and 10 mg/mL). The total volume of each mixture was 1 mL. The microplate was incubated anaerobically for 6 h at 37 °C, and then the mixture in each well was uniformly smeared in a yeast mold agar medium and then cultured at 37 °C for 24 h to check the number of CFUs.

### 2.7. Statistical Analysis

The analysis data were expressed as mean ± standard deviation and were performed in triplicate. One-way analysis of variance (ANOVA) was used to assess significant differences between samples with Duncan’s multiple range test (DMRT) at a 5% level (*p* < 0.05). SAS version 9.4 (SAS Institute Inc, Cary, NC, USA) was used for statistical analyses.

## 3. Results and Discussion

### 3.1. Synthesis of AgNPs

**Figure 1:**
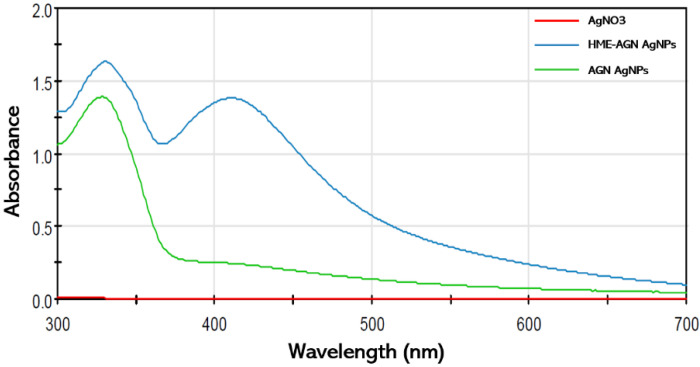
The UV–Vis spectra of AgNPs were synthesized using HME-AGN extract and AGN extract.

The synthesis of AgNPs using HME-AGN aqueous extract and AGN aqueous extract was investigated at 300–700 nm using a UV–Vis spectrophotometer (Figure 1). The dispersion synthesized using the HME-AGN extract showed an absorption band at about 420 nm, but no absorption band was observed when the AGN extract was used. The absorption band at 420–450 nm corresponds to the formation of AgNPs [35], demonstrating that AGN with increased water solubility of the active compound using the HME technique increases the synthesis of AgNPs with higher absorbance intensity.

### 3.2. Visual Inspection

**Figure 2:**
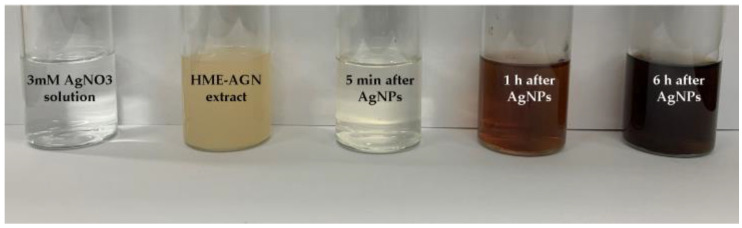
The color change in solution after HME-AGN extract was added to AgNO_3_ solution (5 min, 1 h, and 6 h).

In Figure 2, the formation of AgNPs was confirmed using the AgNO_3_ solution and the aqueous extract of HME-AGN. It was demonstrated that the color of the solution was changed to become dark by the reduction of silver ions, and it was confirmed that as the synthesis time increased, the concentration of AgNPs in the dispersion increased and the intensity of the color increased.

### 3.3. Optimization of Synthesis of AgNPs

Various reaction parameters such as the concentration of the AgNO_3_ solution, the ratio of the extract and Ag, the incubation temperature, and the incubation time were studied for the optimized biogenic synthesis of AgNPs. The UV–Vis spectra were investigated to determine the ideal conditions for the optimal production of AgNPs under various synthetic conditions.

#### 3.3.1. Effect of the AgNO_3_ Concentration

**Figure 3:**
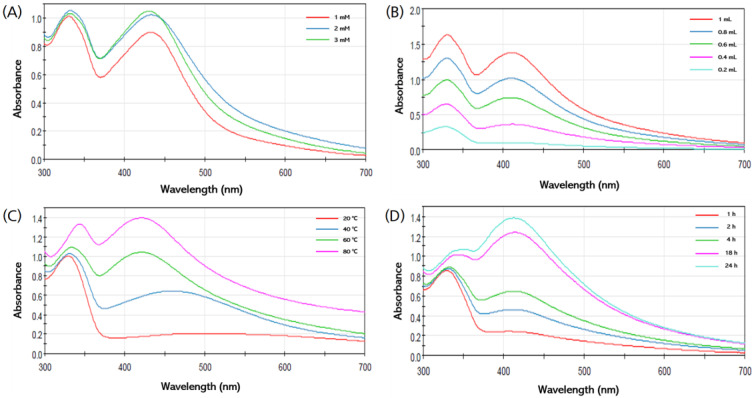
Optimization studies based on UV–Vis spectra of AgNPs synthesized from different (**A**) AgNO_3_ concentrations (1–3 mM), (**B**) HME-AGN extract ratios (1–0.2 mL), (**C**) incubation temperatures (20–80 °C), and (**D**) incubation times (1–24 h).

It was confirmed that as the concentration of the AgNO_3_ solution increased, the synthesis of the AgNPs increased, the silver ions were accumulated, and the color of the solution became darker [10,36]. In order to evaluate the optimal AgNO_3_ concentration for the synthesis of AgNPs, SPR peaks were identified at various concentrations (1, 2, and 3 mM). It was confirmed that the height of the absorbance peak increased as the concentration of AgNO_3_ increased (Figure 3A). Therefore, it was found that the optimal concentration of AgNO_3_ for the synthesis of AgNPs in this study was 3 mM.

#### 3.3.2. Effect of Ratio of HME-AGN Extract

The optimal synthesis ratio was investigated by checking the absorbance peaks according to the ratios of the HME-AGN extract and AgNO_3_ (0.2:9.8, 0.4:9.6, 0.6:9.4, 0.8:9.2, and 1:9 (*v*/*v*)). When the ratio of the HME-AGN extract increased, the intensity of the SPR peak increased and the synthesis of AgNPs increased (Figure 3B). Finally, it was confirmed that the highest AgNPs’ synthesis was obtained when the ratio of the extract to AgNO_3_ was 1:9 (*v*/*v*).

#### 3.3.3. Effect of Incubation Temperature

To confirm the effect of temperature on the synthesis of AgNPs, it was monitored at various temperatures of 20, 40, 60, and 80 °C. As the synthesis temperature increases, the size of the synthesized AgNPs decreases, and as the synthesis temperature decreases, the size of the synthesized AgNPs increases [37]. A broad peak was observed at a low temperature, and it was confirmed that the peak range became narrower as the temperature increased (Figure 3C). The highest and narrowest peak was obtained at 80 °C, indicating the presence of a large number of AgNPs in dispersion.

#### 3.3.4. Effect of Incubation Time

The influence of incubation time on the synthesis of AgNPs was monitored at various time intervals (1, 2, 4, 18, and 24 h). As the reaction time increased, the intensity of the absorption peak increased, confirming that the number of synthesized AgNPs continued to increase (Figure 3D).

### 3.4. Dynamic Light Scattering (DLS) and Zeta Potential Analysis

**Figure 4:**
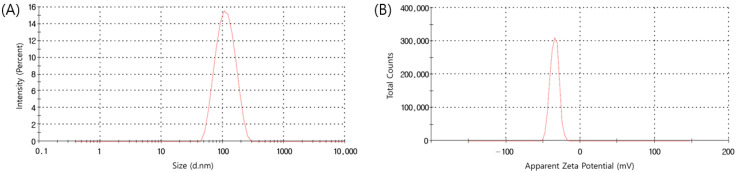
(**A**) Size distribution and (**B**) zeta potential of AgNPs.

As a result of DLS analysis, the average particle size of AgNPs was found to be 102.3 ± 1.35 nm, and the PDI value was 0.314 ± 0.01 (Figure 4A). The closer the PDI value is to 1, the more polydispersed the particle aggregation is indicated to be [38], and when the PDI value is between 0.1 and 0.4, it is considered that the particles are not agglomerated and uniform nanoparticles are produced [39,40]. The zeta potential of the AgNPs depicted in Figure 4B was determined to be −35 ± 0.79 mV. Nanoparticles with absolute zeta potential values greater than 30 mV show a strong repulsive force between particles, which means that they are stable by preventing agglomeration of the particles [41,42].

### 3.5. TEM Analysis

**Figure 5:**
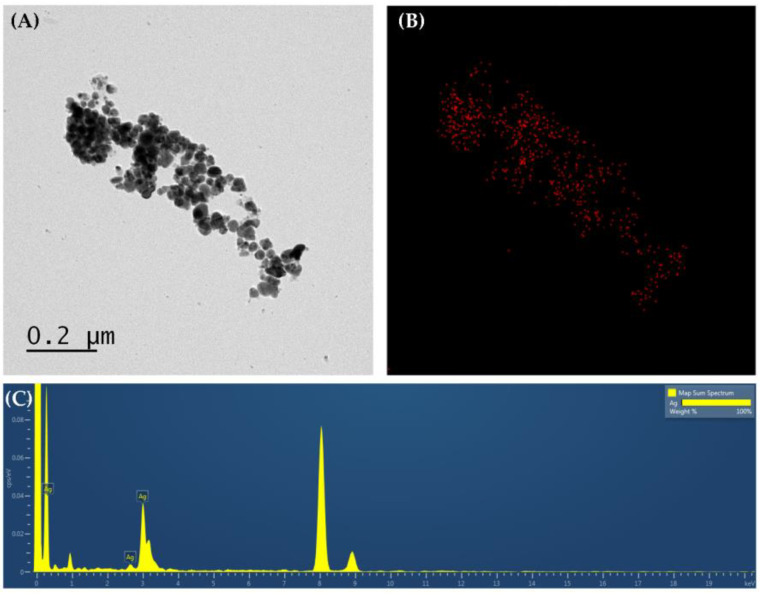
(**A**) Transmission electron microscopic (TEM) imaging of AgNPs, (**B**) EDS mapping of Ag in AgNPs, and (**C**) EDS spectrum of AgNPs.

Morphological characteristics such as the size and shape of the synthesized AgNPs were observed by TEM. The size of the particles was <100 nm and they were found to be polydispersed, and it was confirmed that most of the particles had a spherical shape (Figure 5A). The difference in particle size obtained from DLS and TEM is due to the process related to sample preparation. The particle size measured by DLS is the hydrodynamic diameter where particle size is measured in the hydrated state, whereas TEM is the particle size measured in the dry state of the sample [43,44]. EDS mapping and the chromatogram of AgNPs confirmed the presence of Ag among several elements of the extract (Figure 5B,C).

### 3.6. FT-IR Analysis

**Figure 6:**
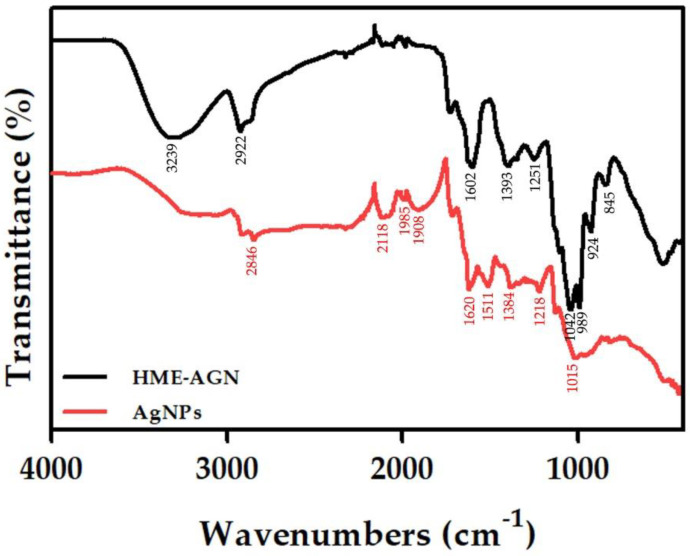
Fourier-transform infrared spectroscopy (FT-IR) spectra of HME-AGN extract and AgNPs.

Figure 6 shows the FT-IR spectrum measured in the range of 400–4000 cm^−1^ to verify information on the interaction with AgNPs synthesized using the HME-AGN extract. The FT-IR spectrum of the HME-AGN extract shows characteristic peaks at 3292, 2922, 1602, 1393, 1251, 1042, 989, 924, and 845 cm^−1^. The peak of 3292 cm^−1^ is the stretching vibration of the OH functional group, the peak of 2922 cm^−1^ is the stretching vibration of C-H, the peak of 1602 cm^−1^ is the stretching vibration of C=O, the peak of 1393 cm^−1^ is the stretching vibration of C=C, and the peaks of 1251 and 1042 cm^−1^ are the stretching vibrations of C-O in alcohol and ether groups. Similarly, the main peaks of AgNPs appeared at 2915, 2846, 2118, 1985, 1908, 1620, 1511, 1384, 1218, and 1015 cm^−1^. The peak of 1602 cm^−1^ in the HME-AGN extract is due to the presence of amide vibration, and the protein was involved as a capping agent and moved to 1511 cm^−1^ in AgNPs. It can bind to Ag ions through cysteine residues and amine groups present in the protein [45]. The appearance of the 1393 cm^−1^ peak in the HME extract and the 1384 cm^−1^ peak in AgNPs may be due to C–H modification and binding of the hydroxyl and carboxylate groups of proteins [46,47]. In addition, it suggests that the -OH group of flavonoids and phenolic compounds present in the extract may affect the stabilization of nanoparticles. Through the results of no significant changes other than a slight shift, it was confirmed that some compounds of the HME-AGN extract played the role of reducing and capping AgNPs.

### 3.7. XRD Analysis

**Figure 7:**
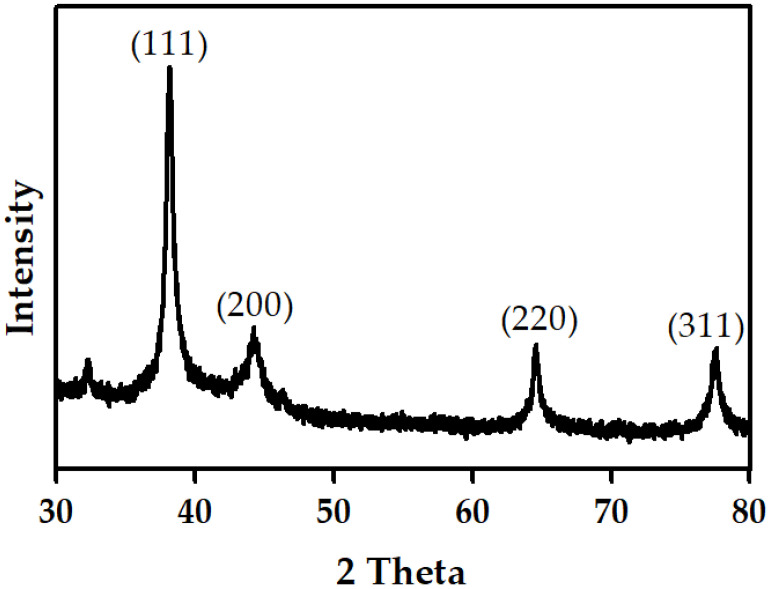
X-ray diffraction (XRD) pattern of AgNPs.

The structural properties of AgNPs synthesized by HME-AGN were studied by XRD at 2 theta angles (Figure 7). The XRD pattern showed major peaks at 38.21°, 44.27°, 64.68°, and 77.59° corresponding to the (111), (200), (220), and (311) planes of Bragg’s reflections, respectively. The diffraction peaks correspond to the face center cubic (fcc) structure of silver, which was compared with the Joint Committee on Powder Diffraction Standards (JCPDS Card No. 04-0783) data [48]. The peak corresponding to the (111) plane is more intense than the other peaks, and is related to the particle size of AgNPs [49,50]. In addition, these peaks are responsible for a strong antibacterial effect [51]. Similar results were also found in the XRD pattern of other AgNPs synthesized using green plant extracts [52,53]. The XRD pattern indicates that the AgNPs formed by the reduction of Ag+ ions by HME-AGN extracts are crystalline in nature.

### 3.8. Antifungal Activity

**Figure 8:**
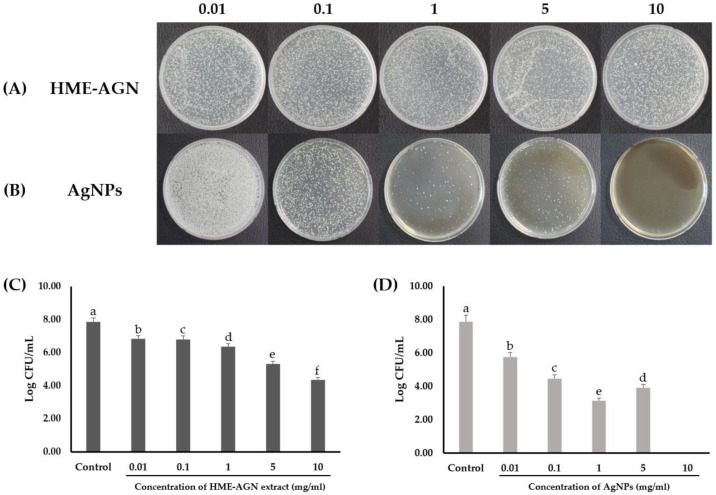
Colony-forming unit (CFU) assays. (**A**) Antifungal activity of HME-AGN extract against *C. albicans*, (**B**) antifungal activity of AgNPs against *C. albicans*, and (**C**) log CFU/mL of *C. albicans* by HME-AGN extracts, and (**D**) AgNPs after 24 h. Data are expressed as means ± standard deviation (*n* = 3). Different letters (a–e) indicate significant differences.

To investigate the antifungal effect on *C. albicans*, the HME-AGN extract and AgNPs synthesized using the HME-AGN extract were treated by concentration (Figure 8A,C). To compare the antifungal effect of AgNPs on *C. albicans* with the HME-AGN extract, the changes in CFU were confirmed by treatment with the same concentration. At the treatment concentration of AgNPs, 0.01 mg/mL showed a log reduction of about 2.12, 0.1 mg/mL was 3.41, 1 mg/mL was 4.73, and 5 mg/mL was 3.96. The plate treated with 10 mg/mL showed a 100% reduction in CFU and showed complete extinction (Figure 8B,D). The result of increased antifungal activity compared to extracts through AgNPs’ synthesis was previously reported [54,55]. The antifungal properties of biosynthesized AgNPs are related to the small size and spherical shape of the nanoparticles, their large surface area, and their permeation to the cell membrane by better contact with the cell wall of bacteria [56,57]. The results shown in Figure 8 suggest that AgNPs synthesized in the HME-AGN extract improved the antifungal activity of the HME-AGN extract.

## 4. Conclusions

In conclusion, when comparing the synthesis of AgNPs using HME-AGN and AGN extracts, it was confirmed that a strong peak was shown at 420 nm when the HME-AGN extract was used. The aqueous solubility of the hydrophobic active ingredient of AGN is increased by HME technology so that it can provide a better reducing agent than that of conventional AGN. The optimal conditions for synthesizing AgNPs were 3 mM AgNO_3_ with HME-AGN extract 9:1 (*v*/*v*), and the highest peak shown in the UV–Vis spectrum was shown at the incubation temperature of 80 °C. It was confirmed that the synthesized particles exhibited a particle size of 102.3 ± 1.35 nm, a PDI value of 0.314 ± 0.01, and a negative charge of −35 ± 0.79 mV. Through the TEM image and EDS mapping, the particle shape was shown to be spherical and the presence of silver ions in the particles was confirmed, and the formation of particles by the reduction of silver ions was confirmed through the XRD pattern. In addition, it was confirmed that the HME-AGN extract and AgNPs acted as a capping agent for the active ingredients of the extract through FT-IR spectrum comparison. Through antifungal studies, AgNPs synthesized using the HME-AGN extract showed a larger decrease in the CFU than the HME-AGN extract, and the decrease in the CFU increased as the concentration increased. In particular, when AgNPs were treated with 1 mg/mL, the highest log CFU/mL decrease was 4.71, and the CFU was not detected after treatment with 10 mg/mL. This study highlights the environmentally friendly synthesis of AgNPs using AGN with increased water solubility using HME, an environmentally friendly process that does not use solvents. In addition to AGN, extracts with increased water solubility through HME can be proposed as a potential alternative for improving the green synthesis of AgNPs and the synthesized AgNPs can be proposed as potential antifungal and therapeutic agents against C. albicans. In addition, for the evaluation of the in vivo treatment of C. albicans by the synthesized AgNPs, studies on the properties of toxicity and the inhibition of biofilm formation should be conducted.

## Data Availability

Not applicable.
